# Study Protocol for Applying Trend Impact Analysis in Health Futures Studies: A Methodological Approach Illustrated by HIV/AIDS Forecasting in Iran

**DOI:** 10.1002/hsr2.70670

**Published:** 2025-04-16

**Authors:** Alikhani Alireza, Hosseini Golkar Mostafa, Sharifi Hamid, Najafi Farid, Haghdoost AliAkbar

**Affiliations:** ^1^ Department of Biostatistics and Epidemiology, Faculty of Public Health Kerman University of Medical Science Kerman Iran; ^2^ Social Determinants of Health Research Center, Institute for Futures Studies in Health Kerman University of Medical Sciences Kerman Iran; ^3^ HIV/STI Surveillance Research Center, and WHO Collaborating Center for HIV Surveillance, Institute for Futures Studies in Health Kerman University of Medical Sciences Kerman Iran; ^4^ Institute for Global Health Sciences University of California, San Francisco San Francisco California USA; ^5^ Research Center for Environmental Determinants of Health, Research Institute for Health Kermanshah University of Medical Sciences Kermanshah Iran

**Keywords:** futures studies, health systems, HIV/AIDS, scenario planning, trend impact analysis

## Abstract

**Background:**

Future planning is critical in the health sector. Data‐driven strategies, such as forecasting, assume that past influential forces will persist under similar conditions. Acknowledging significant events and examining their effects can enhance understanding of potential consequences, improving health system planning. This study elucidates the trend impact analysis (TIA) protocol for forecasting and scenario planning in health systems, focusing on the future of HIV/AIDS in Iran.

**Methods:**

We utilized the TIA approach, which effectively combines quantitative and qualitative methodologies to enhance the precision of forecasting. TIA is specifically designed to account for the influence of unforeseen events and their potential future occurrences on existing trends, thereby facilitating more reliable and comprehensive projections. The methodology uses historical data to extrapolate and forecast future trends quantitatively. Concurrently, qualitative tools identify potential disruptive or influential events that may impact these trends. The approach generates two principal indices: the Impact Rate, which evaluates both the probability of an event's occurrence and the intensity of its effects on existing trends, and the Corrected Estimate Value, which integrates the Impact Rate with the baseline values based on quantitative forecasting. Researchers incorporate events' impacts into the trend projections by systematically analyzing their nature and magnitude. These methodological steps collectively contribute to formulating well‐informed and plausible future scenarios.

**Conclusions:**

The limitations in forecasting the future using quantitative methods and ignoring the effects of unexpected events can lead to “surprises‐free” predictions and cause deviations from estimates and plans. To address this, using innovative approaches in the methodology of studies is crucial. It provides the groundwork for intelligent planning to face the future, ensuring the health field remains dynamic and responsive to change and fostering an optimistic outlook.

## 1 Introduction

In an increasingly complex global environment characterized by rapid technological advancements, heightened communication, and the expansion of virtual spaces, effective planning to address future events and mitigate surprises has become an undeniable necessity. The scope of knowledge and awareness required to prepare for future challenges is vast, making it difficult to keep pace—even with prior preparation [1, 2]. Political, social, technological, and environmental factors have always played a pivotal role in shaping health systems and influencing the future of health policies and plans. Policymakers and managers must proactively identify and respond to these trends to manage their potential consequences effectively [3, 4].

A literature review reveals that most health‐related future research predominantly employs quantitative methods, especially trend extrapolation, as observed during the COVID‐19 pandemic. Numerous studies during this crisis relied on predictive models and data extrapolation to project potential scenarios, highlighting the importance of forecasting in effective planning and preparedness. While this approach has its advantages, it also has notable limitations. Specifically, it often overlooks unprecedented events, weak signals, or “wild cards”—factors that can significantly alter outcomes [5, 6, 7].

These “surprise‐free” results can lead to inaccurate estimates and planning errors by neglecting interactions with trends in other fields and unforeseen events [8, 9]. Thus, reliance solely on trend analysis requires a more comprehensive approach that accounts for a broader range of influencing factors and potential uncertainties. Failure to do so can mislead our understanding of future developments. Analysts frequently use trend analysis to predict national or even global health indicators. However, only a few studies incorporate trend impact analysis (TIA) to systematically factor in the effects of uncertainties. For example, a study on future childhood obesity predicted a prevalence rate of 29.10% by 2031 based solely on quantitative data. However, the inclusion of opposing driving forces suggested a potential reduction in prevalence to 19.57%, whereas positive driving forces could increase it to 32.61%. This example underscores the need to integrate quantitative and qualitative methods across various Futures Studies approaches, including scenario planning [10].

The World Health Organization (WHO) has also emphasized the importance of future research for health planning and development. It has initiated projects focused on health futures research and adopted methodologies for trend and forecasting evaluation [11]. The TIA method offers a holistic approach to studying trends associated with specific issues, considering all probable events, including those of varying intensity, from a forward‐looking perspective [12, 13]. TIA is beneficial in areas where regular data collection and trend monitoring are essential. However, effective future planning also requires expert input and contextual understanding [10]. A systematic search in major databases such as PubMed revealed a scarcity of studies applying TIA, particularly to HIV trends. This gap highlights the pressing need for further research in this area.

Several factors may influence the future of the HIV epidemic. The development of an effective vaccine, the production of new medicines, changes in people's risky behaviors, and dozens of other factors can affect the course of the epidemic. Therefore, more than a simple trend analysis is required to provide sufficient accuracy in defining the future. These problems have received less attention so far, and there are limited discussions on future scenarios of HIV globally and in Iran. The methodologies and activities related to future HIV research vary widely, highlighting the need to enhance methodological knowledge and increase transparency. Developing and publishing diverse scenario planning methods is essential, as these approaches can provide invaluable insights and substantially impact the future of HIV research [14, 15].

This paper aims to explain the TIA methodology for scenario planning within the health system. It uses a case study on HIV/AIDS futures in Iran to illustrate its application and summarize the results.

## 2 Methodological Framework

### 2.1 Study Design

**Figure 1:**
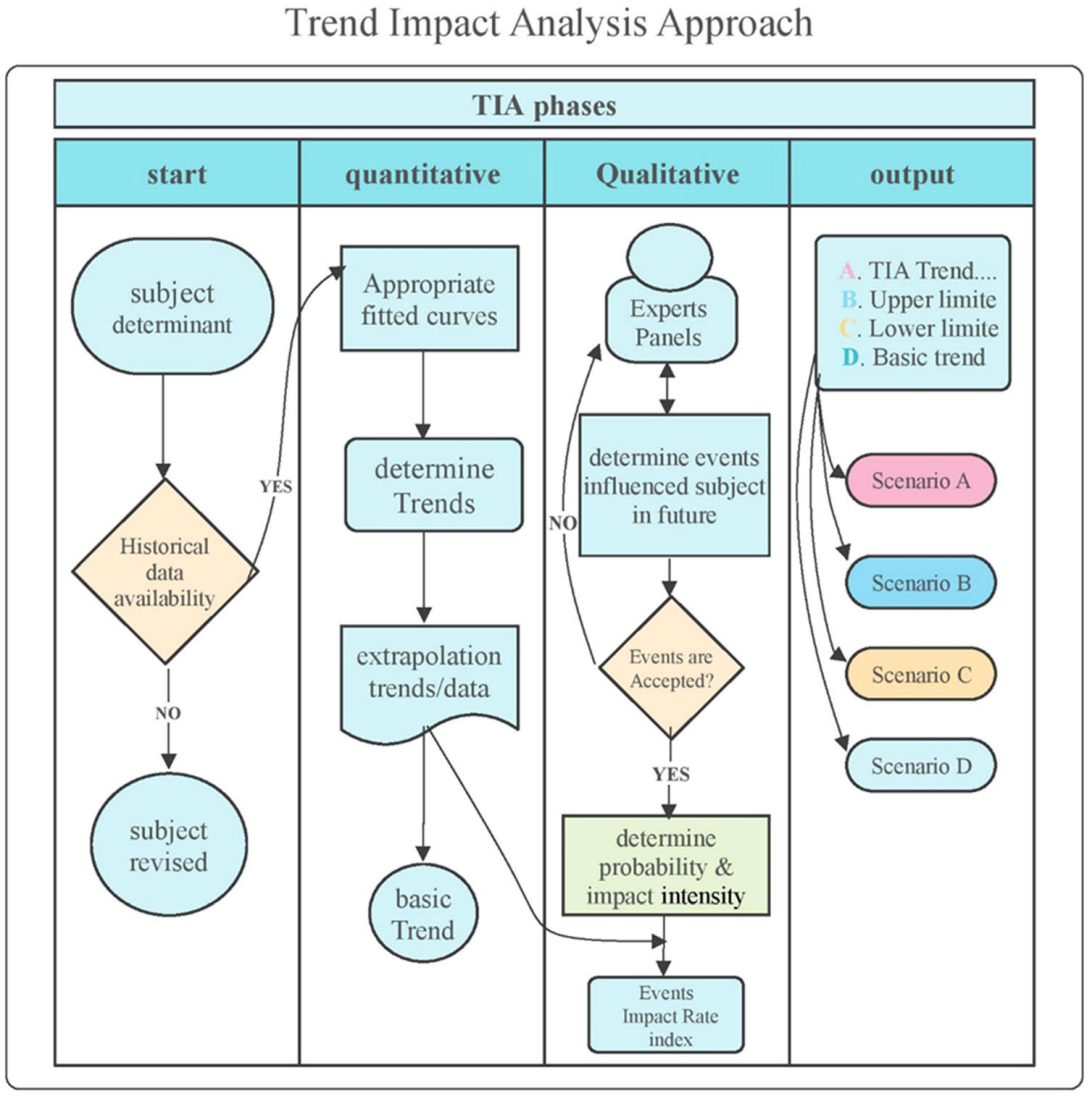
Implementation stages of TIA (*Source:* authors).

The TIA is a suitable approach that integrates quantitative and qualitative methods, serving as a practical tool for scenario writing. Quantitative forecasting methods, such as time‐series analysis and econometric modeling, assume that the forces influencing past data will continue to operate similarly. These methods often fail to account for future events that could disrupt or deviate trends from their expected paths, or they underestimate the significance of such events. Consequently, relying solely on these methods may result in “surprise‐free” predictions and are unlikely to reflect actual future developments. TIA combines the strengths of both quantitative and qualitative approaches to create a novel methodology [16]. After identifying the research subject, the method is implemented in three phases using various tools. Figure 1 provides a general schematic of the implementation phases.

The topic and future horizon we address must be logically justified. For example, HIV/AIDS has always been a priority in the health system, and its incidence and prevalence are crucial from political, social, and health perspectives. The selected time frame aligns with the United Nations Joint Programme on HIV/AIDS (UNAIDS) goal of ending AIDS by 2030 and reducing the annual incidence to fewer than 500,000 cases by 2025 [17]. Therefore, we aim to assess whether these goals are feasible based on current activities, existing indicators, and identified uncertainties. It is crucial to note that the chosen time horizon significantly influences the effectiveness of short‐term or long‐term methods, highlighting the need for strategic planning.

### 2.2 Data Collection

#### 2.2.1 Quantitative Phase

The first phase involves extrapolating the data using historical data and related curve fitting. In this step, we predict future values based on previous data. We used Stata software to apply time‐series techniques in quantitative forecasting based on the available quantitative data and its recording and collection quality. These techniques can help forecast future trends and provide a foundation for strategic planning.

These observations are based on a range of data related to a variable and arranged by time. Quantitative forecasting methods, such as time‐series analysis, aim to forecast the future. Finally, we obtain temporal patterns and identify short‐term, periodic, or long‐term sudden changes in existing trends after describing and explaining the data and presenting its characteristics, such as its upward or downward trend [18, 19]. Time‐series analysis in healthcare services helps predict the outbreak of diseases, the number of patients seeking care, the required personnel in different healthcare departments, and more.

Most quantitative forecasting methods start by determining the general shape of a curve to fit a set of historical data. The forecasting process is rich with variety, as there are different forms of curves for the fit of the data, including linear, polynomial, geometric, exponential, and logarithmic trend curves, each of which can match the historical data well and create different extrapolations. Selecting an appropriate form of the general shape is crucial, and the initial trend line selection is a decision that carries significant weight in the final forecasting [20].

#### 2.2.2 Trend Identification and Forecasting

**Figure 2:**
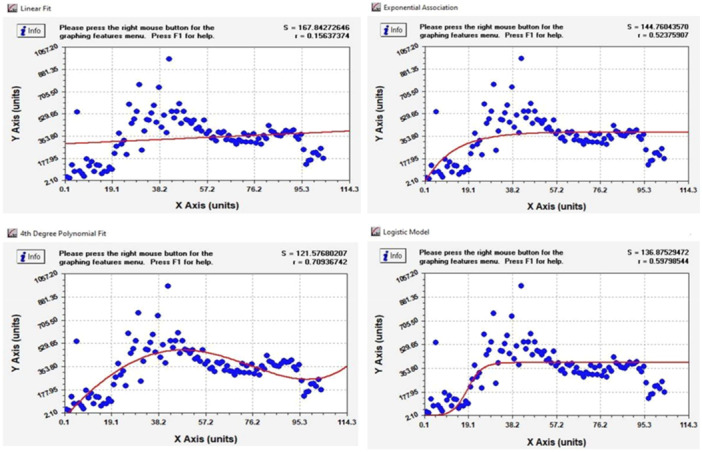
Linear trend line (top left), exponential trend line (top right), polynomial trend line (bottom left), and logarithmic trend line (bottom right), matched with new cases of HIV/AIDS in 25 years (1997–2021).

Using different statistical programs such as Curve Expert, Stata, and R and applying various methods, it is possible to select the most suitable statistical models, make predictions, or draw the best trend lines that match the existing data as closely as possible and achieve better and more accurate extrapolated data. The TIA approach improves preliminary quantitative forecasting, and the more we strive to select a more suitable initial model for the input of the approach, the more the results obtained from the correction of the trends will match reality [21]. Figure 2 shows different trend lines available in Iran to estimate the data concerning the new cases of HIV from 1997 to 2021. Given the charts and the study of existing data trends in identifying new cases, we have drawn four trend lines for data forecasting, and different mathematical and statistical functions can perform the extrapolation.

**Figure 3:**
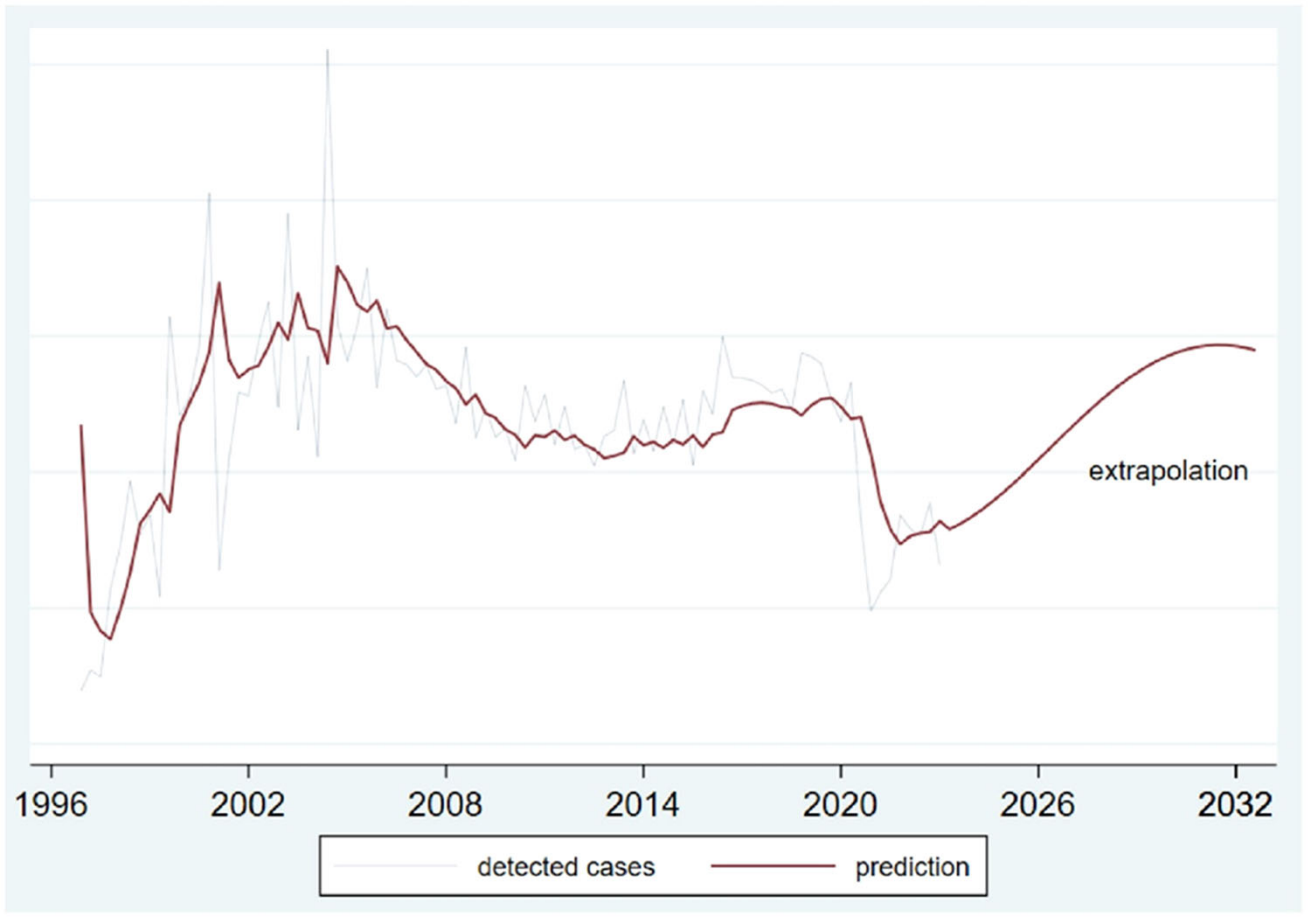
Forecasting and extrapolation trend of identifying new HIV/AIDS patients in Iran during 1997–2021 using the Box–Jenkins model.

The technique of time‐series and technical models such as Box–Jenkins, also known as Auto‐Regressive Integrated Moving Average (ARIMA) models, can be used to achieve a suitable model for forecasting and extrapolating data during the appropriate period in the future. Other models, such as simple regression, multiple regressions, moving averages, and various lesser‐known models applicable to time‐series analysis, can be derived from these models. In addition to the trend factor, seasonal, periodic, and random changes are considered in this modeling method [20]. Figure 3 shows the trend of detected HIV/AIDS from 1997 to 2021 in Iran and forecasts using the Box–Jenkins model up to 2030.

#### 2.2.3 Qualitative Phase

This phase involves compiling a list of influential events that could occur in the future and during the specified period. These events affect quantitative forecasts and cause deviations from existing trends if realized. A mixed‐methods approach is used to tackle complex issues in futures studies in an interdisciplinary field. This approach integrates various methods to address the limitations of individual methodologies. To produce reliable and valid insights for forward‐looking topics that lack sufficient historical data, we simultaneously use data, creativity (visualization), expert knowledge, and collective participation [22].

This step involves engaging relevant specialists and experts based on the criteria for each field. The requirements should align with the research topic and model. Relevant criteria include educational background, experience, publications, and employment in related fields. The case study includes specialists in infectious diseases, epidemiologists, futurists, health managers, field experts, and other professionals such as psychiatrists, social workers, and virologists.

Considering the existing conditions, we can implement this phase using all qualitative tools and methods. These include conducting face‐to‐face interviews, thematic analysis, content analysis, grounded theory, and group‐based methods such as expert panels and the Delphi method. These methods often use inductive reasoning to identify multifaceted interpretations and recurring relationships within social and cultural systems. In this study stage, we utilized two qualitative approaches: content analysis and expert panels. Below is a brief overview of the implementation process for these methods, which clarifies the procedures we followed in analyzing the Identification Driving Forces.

#### 2.2.4 Content Analysis

In our study, we adopted the content analysis approach by incorporating expert opinions and engaging with specialists in the related fields of HIV/AIDS. We also used tools such as face‐to‐face interviews and conducted focused group discussions. The ultimate goal of our content analysis approach was to detect patterns, create coded categories, and identify impactful occurrences. We followed several steps to achieve our objective, which included the following stages:

1. *Defining the research problem and formulating research questions*.
2. *Selecting experts and defining different categories of specialists*.
3. *Establishing a set of coding rules (STEEPV Model)*.
4. *Grouping or categorizing concepts using MAXQDA software*.
5. *Analyzing the data and extracting impactful events or insights*.

#### 2.2.5 Experts Panel

Our aim in employing the expert panel in this study was to network across various scientific disciplines and areas of expertise to achieve convergence and reduce divergence in the information extracted, particularly regarding the key events impacting the future of HIV/AIDS. The different stages of panel implementation, which involved promoting discussions and deliberations among panel members on a set of topics and identified events, were carried out as follows:

1. *The number of areas requiring the formation of panels was determined*.
2. *Experts and specialists were selected for each panel based on their expertise*.
3. *The purpose of forming the panels and the approach to achieving the defined goals were outlined and clarified*.
4. *The scheduling and organization of panel sessions were planned and executed*.
5. *We compiled a final report and documented the key events agreed upon by all panel members*.

#### 2.2.6 Driving Forces Identification

Judgment and imagination are essential elements of the qualitative phase of TIA. In this stage, we must shift from a surprise‐free perspective on the future of trends and consider both necessary and unexpected events [23]. First, we list probable events that should be acceptable, believable, and potentially influential, capable of causing deviations from the extrapolated trends. Various methods, such as environmental scanning, trend analysis, patent analysis, observations, and interviews with experts and specialists, can generate a list of events through a comprehensive review of diverse information sources, ensuring a thorough understanding of potential events [22]. MAXQDA, NVivo, and ATLAS.ti are commonly used software programs for qualitative data analysis. In our study of future AIDS scenarios, we utilized MAXQDA to analyze qualitative data and conduct content analysis, as detailed above.

Driving forces identification will combine the techniques to pinpoint emerging trends and uncertainties. This process will identify factors that may affect future trends in health subjects. For instance, driving forces that may influence HIV/AIDS trends in Iran, such as healthcare reforms, the prevalence of high‐risk sexual behavior patterns, the expansion of online platforms based on sexual services, the integration of artificial intelligence into the healthcare system, and the application of innovations for disease control and therapies, political instability, economic crises and social inequalities, the emergence of drug resistance to current treatments, the occurrence of emerging epidemics, family structure changing, the discovery and access to effective vaccines, the development of stem cell‐based therapeutic techniques, and other potential events. Also, we evaluate the events for their likelihood of occurrence, impact magnitude, and timing.

### 2.3 TIA

TIA will integrate the identified trends and driving forces into baseline forecasts. The TIA approach allows for the correction of time‐series forecasts by considering the potential impacts of emerging trends and events in different situations, including pessimistic and optimistic conditions. The key steps in the TIA process are as follows.

#### 2.3.1 Assess and Apply Driving Forces Impacts

Experts need to provide several indicators regarding the identified events. First, they estimate the probability of each event's occurrence as a function of time, including determining the time from the occurrence of the influential event to the following times:

A. *The trend begins to be affected*.
B. *The impact of the trend will reach its maximum level*.
C. *The impact will reach the final rate or a steady state*.

**Figure 4:**
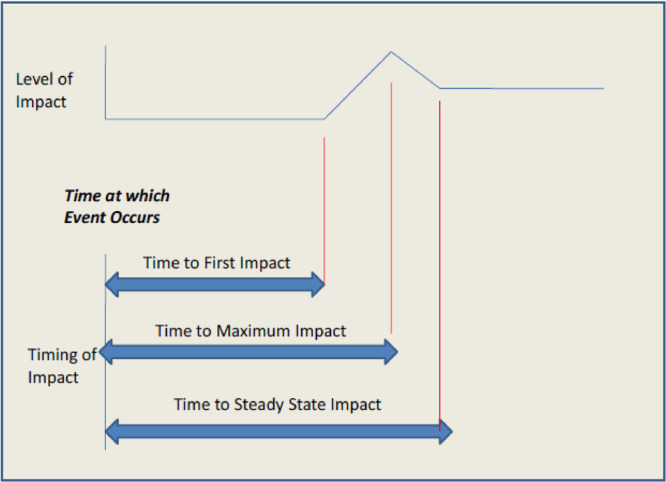
The parameters of the impact of an event in the function of time (adapted from The Millennium Project Future Research Methodology, V3.).

Consider the three determined times and their associated values independently, leading to various potential outcomes. The maximum impact may be positive or negative, while the steady state could reflect no or temporary effects. Sometimes, the maximum impact aligns with the steady state, resulting in sustained effects. The critical aspect is that the impact rate reaches its maximum at a certain point, which could be either positive or negative, and this rate stabilizes at a steady state thereafter [11]. Figure 4 shows the parameters of the impact of an event in the function of time.

The next step in this stage is to determine and estimate the index of impact rate. To calculate the impact rate, use the following formula, which consists of three components:

Impactrate=(a×b)×c

a. *Each event's magnitude or intensity affects existing trends and forecasting models*.
b. *The probability of occurrence of each event in different years within the desired period*.
c. *The numerical baseline of that year according to the forecasts from quantitative models*.

This step is also performed by gathering experts' opinions and converging their views. This step forms an integral part of the process and corrects the extrapolated trends based on the unexpected events likely to happen. After determining these indicators using mathematical methods introduced in the next stage, we will consider the impact of these events on the initial forecast. To study the probability of these events in the following years, experts and specialists should provide their opinions on the likelihood of occurrence, the highest intensity rate, and the time needed to reach a steady state for each new case.

#### 2.3.2 Revise and Develop Forecasts

The final step in this stage involves revising the baseline forecasts by incorporating the impacts of the identified trends and driving forces. Here, we combine all our analyses to form a comprehensive understanding. We then correct the trends and identify the logic of scenarios, a crucial part of our work. The most straightforward approach to dealing with probable identified events is to treat them as independent. Also, one can assume a combined state, where the occurrence of one event influences the probability of another. The cross‐impact analysis (CIA) method is a flexible and complementary approach [24].

To calculate the corrected estimate value, we add the impact rate to the baseline for that year. For instance, the baseline extrapolated trend line for identifying new HIV cases in 2030, based on quantitative studies, predicted 1500 cases. The impact rate of discovering and gaining access to an effective vaccine, with an estimated probability of 50% and a maximum intensity effect of −70%, by the year 2030 is as follows:

Impactrate:(50%×−70%)×1500=−535

In other words, discovering an effective vaccine will reduce the predicted number of cases in 2030 by 525. We will then add this impact rate to the baseline forecast to calculate the corrected estimate value.

Correctedestimatevalue:1500+(−535)=965

**Figure 5:**
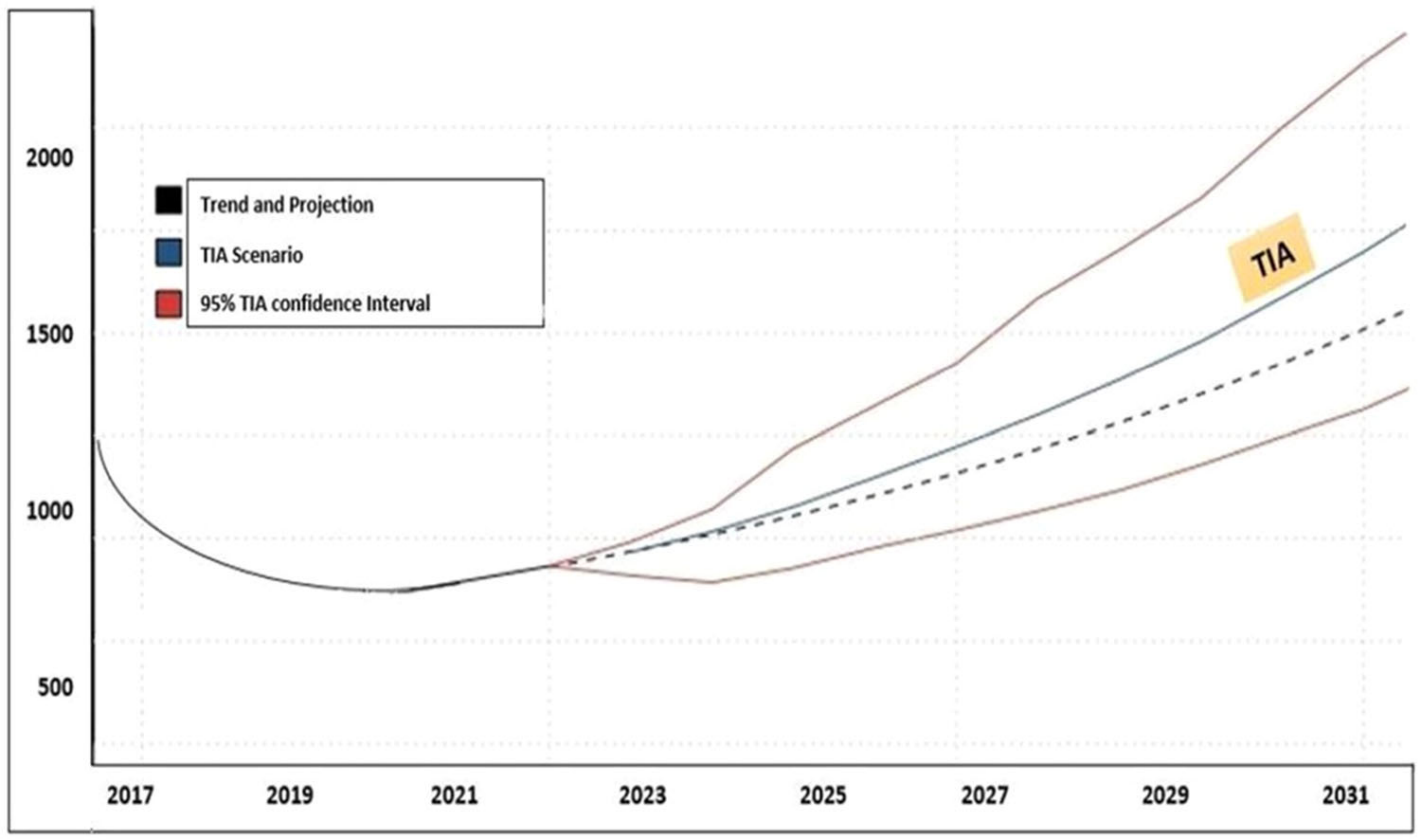
Forecasting the trend of HIV/AIDS patients identified using the Trend Impact Analysis (TIA) approach, adjusted for probable events.

Figure 5 illustrates the output of the TIA approach, showing the estimated number of new HIV/AIDS cases identified up to 2030.

### 2.4 Scenario Development

Identified events, their interpretations, and descriptions of the new situation—represented as trend lines—can serve as scenarios in which different sequences of events occur, and their overall effects will determine the shape of the future curve. As expected, several influential events will be possible in the desired period. In that case, we will apply the sum of all calculated impact rates to the baseline. The outcomes of events' occurrence on the predicted trends can include all possible situations for the highest or lowest impact, which we will mention in different ways.

A solution is to calculate the standard deviation from the mean for the obtained final index. According to the calculation of this confidence interval, the upper and lower limits, as positive and negative scenarios and their median, are considered the corrected trend line resulting from the TIA approach. Another solution is to calculate all the positive direction effects or increases on the existing trend, regard them as the upper limit, and calculate all the negative direction effects, or in other words, the opposite impact on the existing trend, regarding them as the lower limit. The mean will represent the corrected trend line of TIA [16]. In other words, four main trend lines or scenarios emerge. To clarify, we narrated four scenarios using the TIA approach in the study of future HIV/AIDS scenarios in Iran, which include:

- ***Baseline Scenario**: Fluctuation within the cycle of repetition.*
- ***Optimistic Scenario**: Future positive transformations.*
- ***Pessimistic Scenario**: Emerging threats and unknown challenges.*
- ***Synthesis Scenario (TIA)**: Challenges and opportunities along the path of social.*

Another solution is to use the Monte Carlo approach, in which experts provide estimates of the occurrence of certain events in different periods in the future. The sum of these probabilities can reduce uncertainties in decision‐making. In a Monte Carlo simulation, we generate random values for multiple loads across all uncertain parameters in an equation forecasting future values. We define a probability distribution for each uncertain parameter and generate random values, enabling us to calculate the predicted values' upper and lower confidence limits [25].

## 3 Discussion

Researchers have used the TIA technique to a limited extent in the health sector, and no documented or accessible precedent exists for applying this approach specifically to HIV/AIDS. Most studies forecasting HIV/AIDS have mainly relied on trend analysis and statistical modeling based on historical data without considering weak signals and the impact of critical events. Such as the Prevalence of HIV in Kazakhstan 2010–2020 and its forecasting for the next 10 years [26], Forecasting future HIV infection cases: evidence from Indonesia [27], and Forecasting of HIV/AIDS in South Africa using 1990–2021 data [28]. They use statistical ARIMA models and another mathematical model to forecast the HIV infection prevalence rate and newly infected rate.

In contrast, our study introduces the TIA method to HIV/AIDS forecasting and incorporates it into forecasting trends. The TIA approach allows us to identify eight positive and five opposing directions as driving forces and develop probable and plausible scenarios by applying impact rates to extrapolated trends. Finally, we ensured that the forecasts considered the influence of future events, creating a more realistic and flexible set of predictions for HIV/AIDS future trends in Iran.

Several other studies have demonstrated the potential of TIA to improve forecasting accuracy and support decision‐making. For example, researchers conducted a community‐based TIA study on childhood obesity in Iran. They identified two positive and five opposing driving forces. The quantitative forecast for 2031 initially estimated the prevalence of overweight and obesity at 29.1%. However, the TIA scenario, which accounted for the influence of positive and negative drivers, projected a lower prevalence of 23.07% [10].

In the study, researchers examined scenarios of the impacts of the COVID‐19 pandemic on socioeconomic conditions and public health. They developed scenarios based on probable events and directed policymakers' attention to driving forces and trends. Their findings underscored that neglecting these factors could lead to severe consequences, including increased mortality, the collapse of the national healthcare system, and economic and social crises [29].

Another study, conducted outside the health sector, applied the TIA to explore the future of oil prices and influencing factors. The results indicated that TIA‐based estimations were more aligned with real‐world outcomes, and the trend‐adjusted forecasts using this technique led to improved quantitative predictions [21]. Other applications of TIA include forecasting housing prices by incorporating key influencing factors and variables [30], technology forecasting and wheat farm performance scenarios in India [31], and the application of TIA for predicting future fruit consumption [32]. These studies confirm that TIA can refine extrapolated projections, providing reliable and actionable forecasts.

The essential strength of TIA is its thorough approach to investigating different future events. Rather than simply accepting and applying a trend of past changes, this method incorporates insights from related experts, examines the probabilities and effects of events, and provides suggestions. The study of events explores deeper layers and influential partial factors for each variable, ensuring consideration of all relevant factors. This approach can quantify the scenarios and make them objective, increasing confidence in internal consistency. Moreover, it explicitly considers uncertainty in decision‐making analyses by providing a confidence interval or range rather than a one‐dimensional prediction, aligning with risk‐analysis techniques.

On the other hand, some limitations should also be acknowledged. Among them, we cannot be certain that we have achieved a complete list of unpredictable events or be entirely sure of the accuracy of judgment and probabilities of the events' effects because such lists of events are only speculations about the future that may be true or false. Nevertheless, using approaches such as TIA and developing scenarios resulting from this approach can help better understand and logically deal with the future of health‐oriented programs, improving the decision‐making process and health program management.

## 4 Conclusion

This study demonstrates the application of the TIA approach to forecasting and scenario planning within health systems. This study demonstrates the application of the TIA approach in forecasting and scenario planning within health systems. During the TIA stage, two critical indicators, the impact rates of events and the corrected estimate values, were extracted, enabling the revision and refinement of the predictions. Ultimately, four potential scenarios were determined: the continuation of the current trend, a pessimistic scenario driven by negative triggers, an optimistic scenario driven by positive triggers, and an integrated scenario (TIA), which combines all relevant factors.

By identifying and applying the impact rates of plausible events on the forecasted trends, we developed alternative scenarios that improve the integration of historical data and expert opinions from relevant scientific fields. The results refine the predictions generated through modeling methods, enhancing their reliability and utility for policymakers. This paper emphasizes the importance of employing flexible, integrated methodologies in health futures studies to support informed decision‐making in managing health‐related challenges.

## Author Contributions

**Alikhani Alireza:** writing – review and editing, conceptualization, methodology, formal analysis, writing – original draft. **Hosseini Golkar Mostafa:** methodology, writing – review and editing. **Sharifi Hamid:** supervision, funding acquisition, writing – review and editing. **Najafi Farid:** writing – review and editing, supervision. **Haghdoost AliAkbar:** funding acquisition, supervision, writing – review and editing, writing – original draft.

## Conflicts of Interest

The authors declare no conflicts of interest.

## Transparency Statement

The lead author, AliAkbar Haghdoost, affirms that this manuscript is an honest, accurate, and transparent account of the study being reported; that no important aspects of the study have been omitted; and that any discrepancies from the study as planned (and, if relevant, registered) have been explained.

## Data Availability

The data that support the findings of this study are available on request from the corresponding author. The data are not publicly available due to privacy or ethical restrictions.
